# Soldering of Passive Components Using Sn Nanoparticle Reinforced Solder Paste: Influence on Microstructure and Joint Strength

**DOI:** 10.3390/nano9101478

**Published:** 2019-10-17

**Authors:** Anas M. Atieh, Tala J. Abedalaziz, Abdulaziz AlHazaa, Michael Weser, Wael G. Al-Kouz, Maen S. Sari, Ibrahim Alhoweml

**Affiliations:** 1 Industrial Engineering Department, School of Applied Technical Science, German Jordanian University, Amman 11180, Jordan; 2 Würth Elektronik eiSos GmbH & Co. KG, EMC & Inductive Solutions, Max-Eyth-Str. 1, 74638 Waldenburg, Germany; 3 Research Chair for Tribology, Surface, and Interface Sciences, Department of Physics and Astronomy, College of Science, King Saud University, Riyadh 11451, Saudi Arabia; 4 King Abdullah Institute for nanotechnology, King Saud University, Riyadh 11451, Saudi Arabia; 5 Mechatronics Engineering Department, School of Applied Technical Science, German Jordanian University, Amman 11180, Jordan; 6 Mechanical Engineering Department, College of Engineering, Prince Mohammad Bin Fahd University, Al-Khobar 34754, Saudi Arabia; 7 Mechanical and Maintenance Engineering Department, School of Applied Technical Science, German Jordanian University, Amman 11180, Jordan

**Keywords:** nanocomposite solders paste, reflow soldering, solder reliability, soldering CT scan

## Abstract

In this study, the effects of adding Sn nanopowder (particle size < 150 nm) to three solder pastes SAC3-X(H)F3+, SCAN-Ge071-XF3+, and water washable WW50-SAC3 are evaluated regarding microstructure, morphology, joint strength, and electrical resistance. The nanopowder was added at a rate of 10% by weight and then mechanically mixed until homogenous solder paste was obtained. The results showed that the addition of Sn nanoparticles resulted in homogenous bond formation for SAC-3 and SCAN, while voids and bubbles formation slightly increased within the joint interface for the water washable solder paste. The SCAN + Sn nano reinforced solder paste showed increased variation of joint strength from 12.6 to 39.9 N, while the water washable + Sn nanopowder reinforced solder paste showed less variability in joint strength from 17.3 to 33.9 N. Both sets of solder paste with and without Sn nano reinforced solder paste showed a reliable quality joint under mechanical shock testing after six shocks in six milliseconds with an 87.1 ms pulse duration. The results showed that Sn nanoparticles resulted in a small resistance change, while RDC values (in mΩ) slightly decreased for SAC and increased for SCAN and further increases for water washable solder paste.

## 1. Introduction

**Figure 1:**
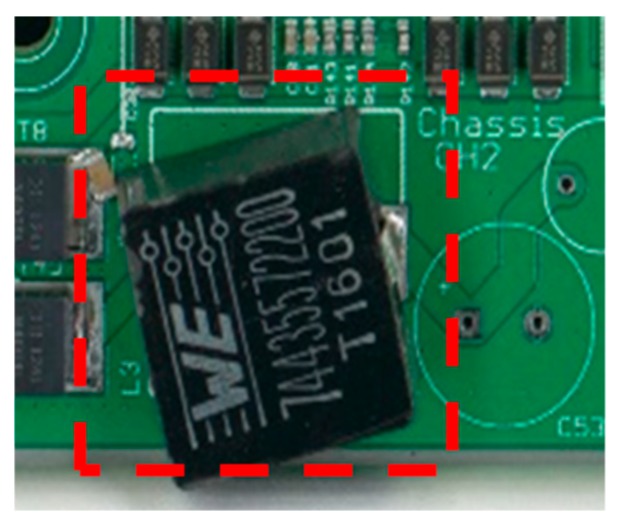
Sample for moving passive component after soldering.

Since the international legislation, EU RoHS, that banned the lead-based solder paste due to environmental and health toxicity of lead, extensive research and development have been carried out for the development of a suitable, industrial grade, lead-free solder paste [1,2,3]. One of the solders that have been used extensively in electronic packaging applications is tin-based lead-free solders [4,5,6,7]. Different alloying elements were utilized with the tin-based solders, such as Ag, Ag-Cu, and Cu. However, it was reported in the literature that extensive research is still needed to overcome industrial challenges and to increase the service quality of those solders, such as high melting temperature compared to the lead-based solders that may lead to higher thermal stresses. Intermetallic compound (IMC) formation within joint regions that include Sn dendrites and large Ag_3_Sn and long Cu_6_Sn_5_ resulted in deterioration of joint properties and caused easy oxidation, localized strain, and poor wettability. Furthermore, Zhang and Tu reported the need for high creep and thermal fatigue resistance lead-free solder pastes. They have also reported that the addition of nano and microparticles to the solder pastes is still academic, with minor industrial applications reported [8]. The industrial need differs from the lab development in different aspects; for instance, while joint strength increase is the target for lab work, it is the electrical conduction for industrial-based applications. Furthermore, the industrial use of Sn-based solder paste includes a few challenges, such as high melting temperature, difference in thermal efficiency, and poor joint formation [4]. Previous work related to the addition of nanoparticles to solder pastes showed that different nanopowder could be utilized for joint production optimization [1,2,8,9,10,11,12,13,14]. Chellvarajoo et al. studied the effect of the addition of Fe_2_NiO_4_ nanopowder to Sn-3Ag-0.5 Cu solder paste, and it was reported that IMC thickness was reduced up to 59% after adding 2.5% Fe_2_NiO_4_ nanopowder to Sn-3Ag-0.5 Cu, while nano-indentation showed more than double the hardness value [9]. In their other study, Chellvarajoo and Abdallah utilized NiO ceramic nanoparticles to produced unique Pb-free nanocomposite solder paste. The addition of this ceramic nanopowder, at the same rate as in their other study, resulted in a 50% IMC thickness reduction [4,10]. Amagai studied the effect of 11 different nanoparticles addition to Sn-Ag lead-free solder paste on the hardness, grain size, and IMC thickness. It was found that Co, Ni, or Pt inclusions in Sn–3Ag based lead-free solders did not increase IMC thickness nor grain size significantly. On the other hand, Al, P, Cu, Zn, Ge, Ag, In, Sb, or Au inclusions in Sn–3Ag based lead-free solders increased IMC thickness after four solder reflows [11]. From an industrial point of view, melting temperature depressant is considered in favor of two means; less cost and less temperature tolerant printed circuit boards (PCBs) [1]. However, careful selection of nanoparticles is needed to improve solder mechanical properties, where a nanocomposite reinforced solder joint is produced [2,13,14,15,16,17]. It was reported that decreasing the average size and distance between intermetallic compounds enhance the microstructure, where the Sn-Cu solders consist of βSn, eutectic Sn-Cu, and Cu_6_Sn_5_ intermetallic compounds [2]. It was reported to the Würth Elektronik eiSos technical department that an electronic component did not fully solder on the circuit board and resulted in a problematic movement of the component after soldering in a reflow oven. It was speculated that there is a relation between component type and solder paste used that resulted in this malfunction. Hence, the aim of this research work is to investigate the effect of solder-paste material and the addition of Sn nanopowder on the soldering of passive components on printed circuit boards (PCBs). This work focusses on the effect of different solder paste types on the microstructure and joint strength. Figure 1 shows an example of the movement of a passive component after soldering. To the best of authors’ knowledge, no research has been published which has investigated the effect of Sn nanopowder on the microstructure and mechanical properties of SCAN-Ge071-XF3+, SAC3-X(H)F3+, and water washable WW50-SAC3 solder pastes. Hence, as per the key review paper of Zhang and Tu [8], that the research on adding micro and nanoparticles to lead free solders is still academic because no applications in real solder joint technology are found nor any commercial nanoparticle solder paste, this paper took the extra step of researching the industry application of nanoparticle reinforced solder paste. 

## 2. Methodology

The tested component in this research work is a WE-CNSW component, which is commonly used in USB applications, for application in High-Speed Data Lines, Common Mode Noise Sources, and LAN. It has a size of 0805 mm and is comprised of either tin or gold plating surfaces. These components were visually tested thoroughly to deduce notable differences between them.

**Figure 2:**
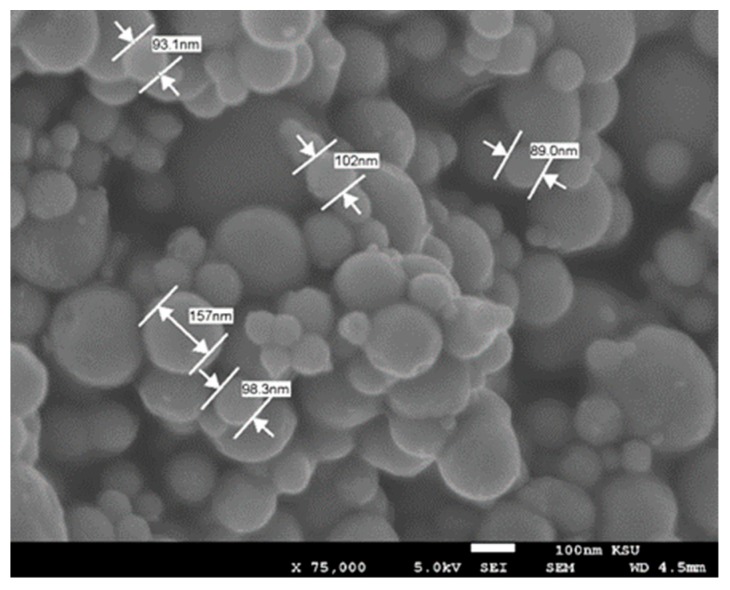
Sn nanopowder.

The influence of three types of solders was compared; namely; SCAN-Ge071-XF3+, SAC3-X(H)F3+, and water washable WW50-SAC3. Each one of those pastes consists of a solder alloy, paste, and flux content, and all were bought from BalverZinn Company, Germany. In addition to these three standard pastes, a tin nanopowder (Sn, particle size < 150 nm, 99% pure) was bought from Sigma Aldrich, Germany. Figure 2 shows the Sn nanopowder.

**Figure 3:**
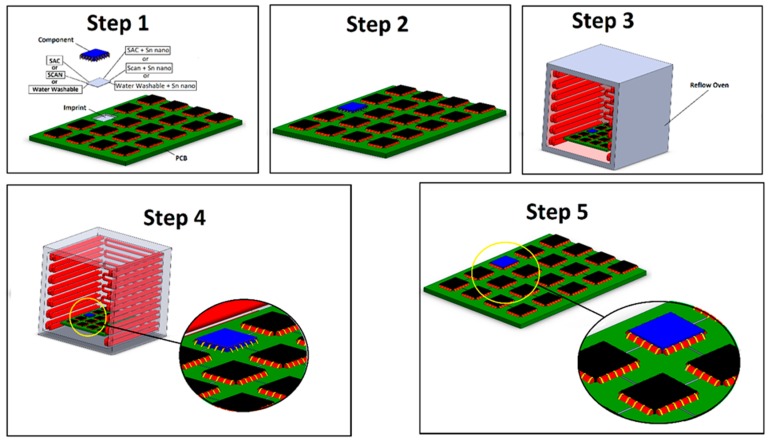
Illustration for soldering steps for components on printed circuit boards (PCBs) using different solder pastes.

The nanopowder was added at a rate of 10% by weight (each solder was used as it is, as well as mixed in a 10:1 mass ratio with Sn nanopowder). Hence, six groups of testing samples were created, and at least 20 components from each group were produced for testing. The SCAN-Ge071-XF3+ is a no-clean paste and it prints more clearly in comparison to the water-soluble paste due to the resin that it contains. The resin maintains the shape after printing and has excellent oxidation barriers during the reflow process, while the SAC3-X(H)F3+ is a no-clean solder paste. The WW50-SAC3 works because of a soluble reaction, making it highly active and efficient at removing oxides; however, the resin is not very soluble in water washable solder paste and this paste does not print as clearly as the no-clean solder pastes. The chemical compositions of the three pastes tested are shown in Table 1, and Figure 3 shows the soldering steps.

The experimental steps include first applying the solder paste by printing on the PCB and then adding the WE-CNSW components. However, before moving this setup into the reflow oven (model Solano DIMA SMT systems RO-500), visual inspection was done to make sure no movements occurred and to readjust the component to the correct form and to ensure that the pad size and tolerances were relative to their placement. If this was not done, it would be difficult to detect and fix the component issues until they were out of the oven. In some cases, the vibration of the reflow oven might cause the components in the PCB to shift from their position during soldering process, hence we used an automatic Pick and Place machine (DIMA SMT system ATOZ PP-050), which uniformly aligns all the components under the same pressure and temperature settings, and prevents them from shifting in the reflow oven.

The soldering process was conducted for all lead-free solder pastes SAC, SCAN, and water washable at a temperature of 235 °C, utilizing a four step process within the reflow oven. The stages were preheating, soaking, peak, and cooling. In the first stage, the PCB and components were heated to +3 °C/s to maintain even heating between the PCB and component by activating the flux in the solder paste. In the second stage, the time and speed of the oven were set with consideration of the component size so that the solder paste could dry sufficiently. The temperature was quickly raised in the third stage above the liquid melting point of the solder pastes. The fourth stage allowed for the cooling down process resulting in solder solidification.

A cross-section of the components was produced by mounting the samples, cutting, and then grinding and polishing with different paper grits until reaching a mirror-like surface on a semi-automatic Buehler Alpha AutoMet 250 grinder/polisher. Microscope Keyence VHX5000 (industrial grade with 54 Megapixel 3CCD) was used to ensure dimensional accuracy, physical measurements, and cross-sectional analysis. Then a JSM-7600F scanning electron microscope (SEM) (JEOL, Tokyo, Japan) was used to study and characterize the microstructure of the joints. The cross-section was also used to test if the meniscus was in good shape to determine if the thickness of the solder paste was suitable, to ensure that there were no voids or cracks, and to ensure that there were no gaps in the components.

A Computed Tomography (CT) machine with an X-ray device (model GE phoenix nanotom) was used to generate 3D images generated from 1400 images for each component in order to build a complete 3D model. Push and pull testing were also performed to measure the joint strength according to the Automotive Electronics Council (AEC) standard AEC-Q200 (calibration certificate according to ISO7500-1), which is a full computer-controlled programmable machine generating force between 0.1 N to 200 N at a crosshead speed of 1 mm/min.

Finally, vibration tests up to 6 Hz and mechanical shocks up to 100g were performed using the shaker system V780. This test was conducted to check if the soldered components could fall off during severe service conditions of the PCB due to bad soldering.

## 3. Results and Discussion

### 3.1. Microstructural Analysis

**Figure 4:**
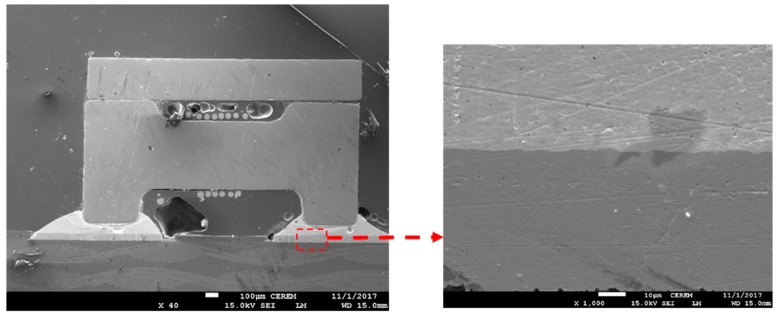
SEM micrograph for the joint interface of a passive component with SAC solder paste.

**Figure 5:**
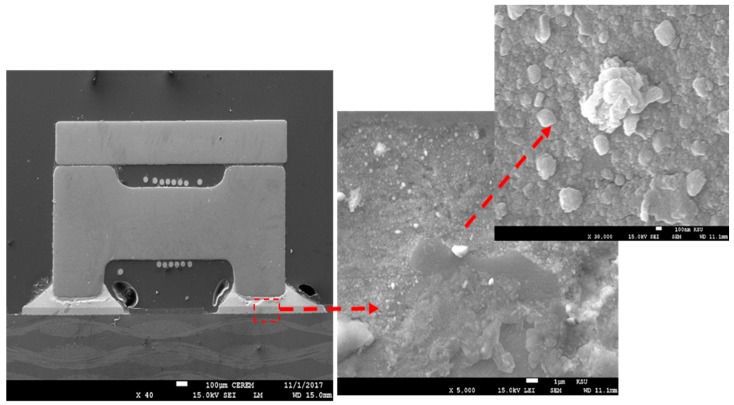
SEM micrograph for the joint interface of a passive component with SAC-nano solder paste.

**Figure 6:**
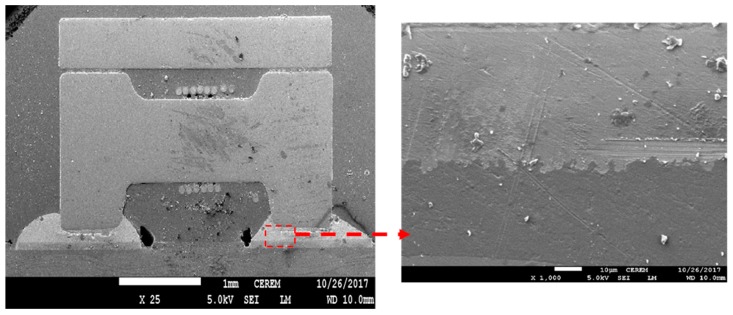
SEM micrograph for the joint interface of a passive component with SCAN solder paste.

**Figure 7:**
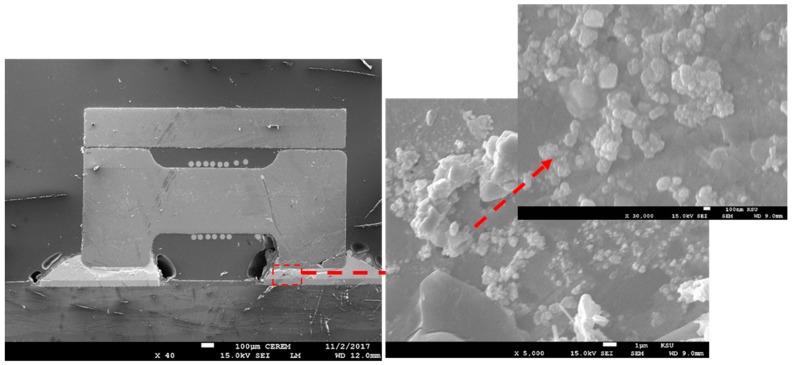
SEM micrograph for the joint interface of a passive component with SCAN-nano solder paste.

**Figure 8:**
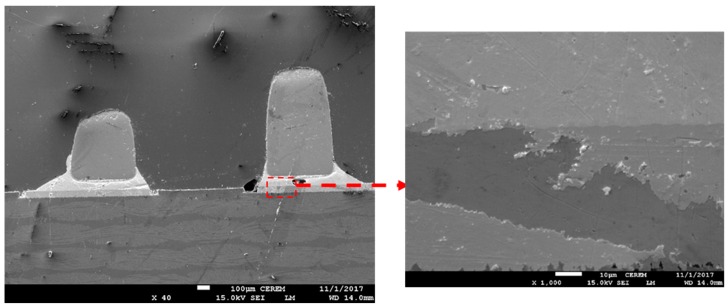
SEM micrograph for the joint interface of a passive component with water washable solder paste.

**Figure 9:**
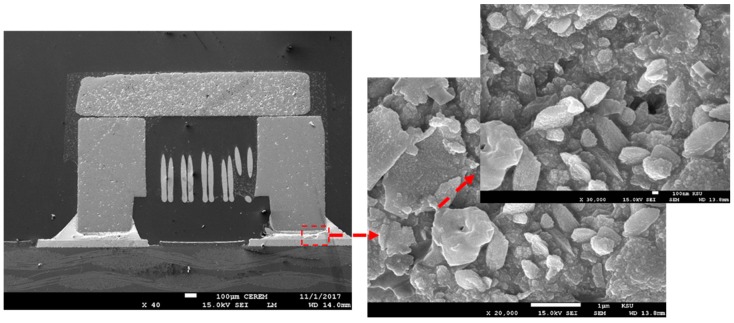
SEM micrograph for the joint interface of a passive component with water washable-nano solder paste.

The results of SEM analysis for the tested components as a function of different solder paste with and without Sn nanopowder are shown in Figure 4, Figure 5, Figure 6, Figure 7, Figure 8 and Figure 9. Homogenous joint interfaces were observed for both SAC and SCAN solder paste with and without nanopowder, while in the water washable paste, some microcracks and voids were observed. However, differences in surface morphology were also observed among the three tested solder pastes. Figure 4 and Figure 5 show the joint interface of PCB soldered with SAC paste, and it was observed that in both cases with and without Sn nanopowder, the joint interface was straight and horizontal, which indicates that bonding kinetics occurred at the same rate all over the joint interface. No bubbles or voids were observed within/on the joint interface.

With regards to the SCAN solder paste, a lesser degree of homogeneity was observed in both cases with and without the Sn nanopowder reinforcements. A non-uniform joint interface morphology was observed, where even though no straight-line interface was observed in the SCAN paste samples, no voids were seen within the joint interface. While in the SCAN-added Sn nanopowder samples, some minor voids were observed on the joint interface with the same behavior of non-uniform morphology. However, more flow of the component base materials was observed towards the SCAN Sn nanopowder joint side. This irregularity of joint interface in the case of SCAN-soldered samples indicates that joint formation kinetics may occur at different rates across the joint interface (see Figure 6 and Figure 7).

Figure 8 and Figure 9 show the SEM micrograph of the joint interface of the passive component and water washable solder paste. The horizontal interface was observed, and evidence of some voids was observed in both samples with and without the addition of Sn nanopowder. Bubbles, interestingly, were observed in higher concentrations all over the water washable solder paste whenever the Sn nanopowder was used.

### 3.2. CT Scan Analysis

**Figure 10:**
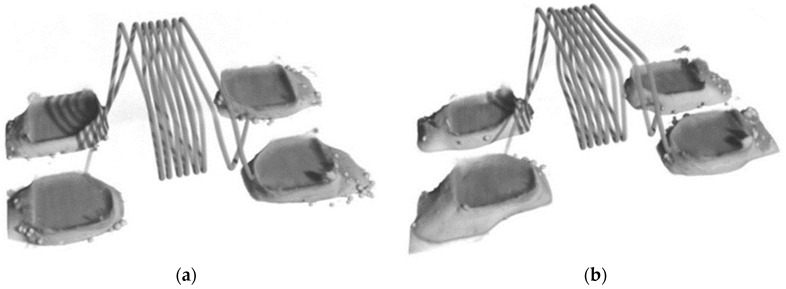
3D CT scan of the component with (**a**) SAC solder paste and (**b**) with Sn nano reinforced SAC solder paste.

A CT scan test was done from every solder paste to see if the solder paste was soldered completely in the reflow oven or not. A 3D model was generated from the 1400 images taken for each soldered sample. Pure SAC solder paste was the only paste between the other two pastes that had bubbles in the CT test, even when no nano paste was included, as shown in Figure 10a.

Nano SAC solder paste was the only paste between the nano pastes that showed a little number of bubbles. The bubbles mean that more time is needed to solder it and it is not completely soldered as shown in Figure 10b.

**Figure 11:**
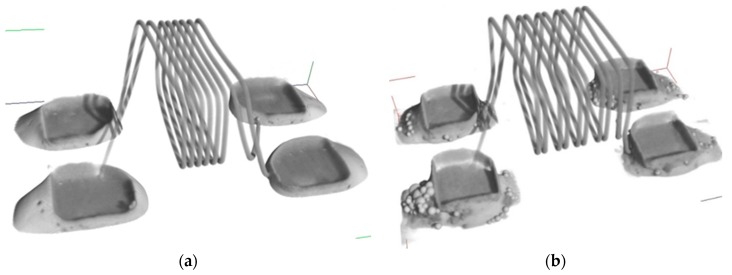
3D CT scan of the component with (**a**) SCAN solder paste and (**b**) with Sn nano reinforced SCAN solder paste.

Figure 11a shows the SCAN solder paste performance; it was observed that the resulted joint thickness is thinner, and it is contributed to the printing method. Figure 11b shows the CT scan for the nano SCAN solder paste, where excessive amount of paste still exists, which means more time is needed to complete the soldering process, which also might suggest that the temperature of the reflow oven was not enough for Sn nano SCAN paste to dissolve, as shown in Figure 11b.

**Figure 12:**
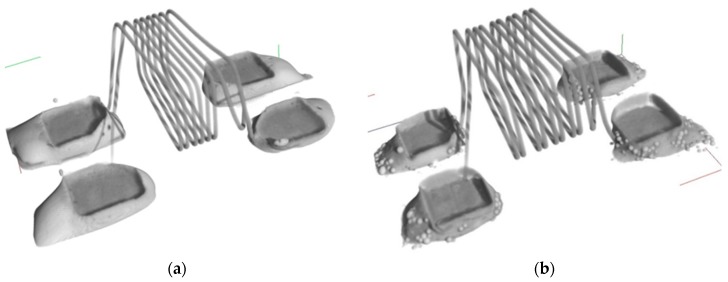
3D CT scan of the component with (**a**) water washable solder paste and (**b**) with Sn nano reinforced powder water washable solder paste.

The water washable solder paste showed complete homogeneity with no defects, as seen in Figure 12a. However, the nano paste water washable solder paste showed that it needs more time to solder completely, as shown in Figure 12b for the water washable mixed with Sn nanopowder paste. When the temperature is heated on the solder paste and flux is activated, the flux removes the oxide films from the surfaces and, therefore, the joint occurs. The more oxides that exist on the solder particles, the longer it takes, and more voids will remain at the end. Therefore, it is advisable to give enough time for the flux to remove the oxides.

### 3.3. Evaluation of Joint Strength 

**Figure 13:**
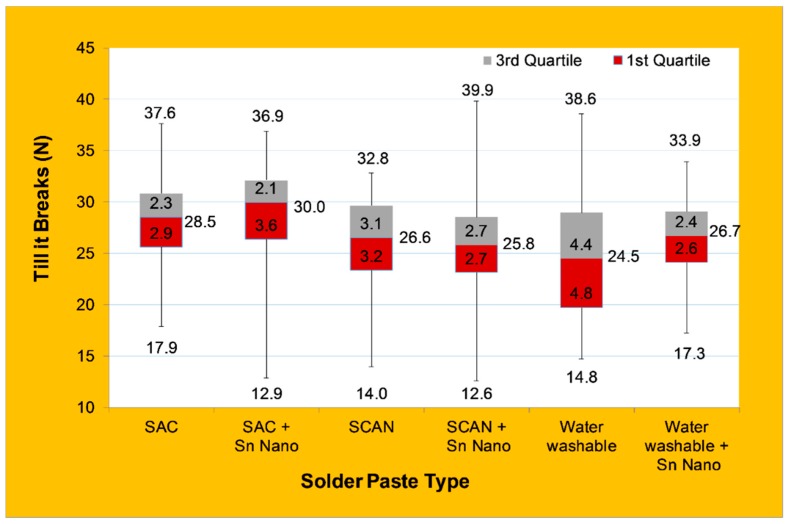
Effect of solder paste type on joint strength.

Sixty components from each solder paste type were tested for strength. The results of the maximum, minimum, average, and quartiles of wired components are shown in Figure 13. 

The SCAN reinforced Sn nanopowder joint showed the lowest strength of 12.6 N among all tested samples. Furthermore, the SCAN solder paste showed the lowest solder paste strength of 14.0 N among the non-added Sn nanoparticles pastes. It is observed that the addition of nanopowder to SCAN paste resulted in enhancing the test variations, slightly reducing the average strength, and improving the maximum strength from 32.8 to 39.9 N. 

Even though the addition of Sn nanopowder to SAC solder paste slightly enhanced the average strength compared to SAC without nanopowder, it resulted in increasing the variations of the test results. The results showed that it lowered the maximum achievable strength to 36.9 N. The addition of nanopowder to SAC paste also resulted in enhancing the test variations, which coincide with the observation for the SCAN reinforced Sn nanopowder. The reverse effect was observed for water washable solder paste, where the addition of Sn nanopowder resulted in increasing the average strengths but reduced the maximum achievable strength from 38.6 N to 33.9 N. Furthermore, the addition of nanopowder reduced the test variations. 

Although improving the mechanical strength of the joints is a critical objective for research and development projects, it is not a target in the soldering industrial applications. As for the flip-chip technology of electronic packaging, the joints produced are required to have a low mechanical strength so it can yield easily due to the processing issue of chip-packaging interactions [8]. As the size of the silicon chips decreases, the need for optimization of the joint strength increases, but then a stronger joint is not necessarily required [8]. The softer effect seen before and during the use of lead-based solder paste through the presence of Pb was not seen in this work. In our research of Sn nanoparticle reinforcement solder application in the aforementioned solder paste, the addition of Sn nanoparticle paste only slightly affects the average joint strength, which suggests the suitability for the electronic packaging industry. 

### 3.4. Mechanical Shock

The standard test of mechanical shock was conducted to examine the solderability of components on the PCB’s during severe service conditions. This test aimed to identify whether solder joints of WE-CNSW components assembled on the PCB would break when the latter was subjected to dynamic and heavy bending during the process, packing, shipping, and daily use. Therefore, this test was made on all the PCB’s after soldering both with and without Sn nano reinforced paste to check if the components would fall down after six shocks in six milliseconds on the three axes of the PCB (X, Y, and Z). The pulse duration lasted for 87.1 ms, with a 100 g acceleration peak and a velocity change of 1.91 m/s, while the acceleration was from −149.9 m/s^2^ to 1000 m/s^2^.

**Figure 14:**
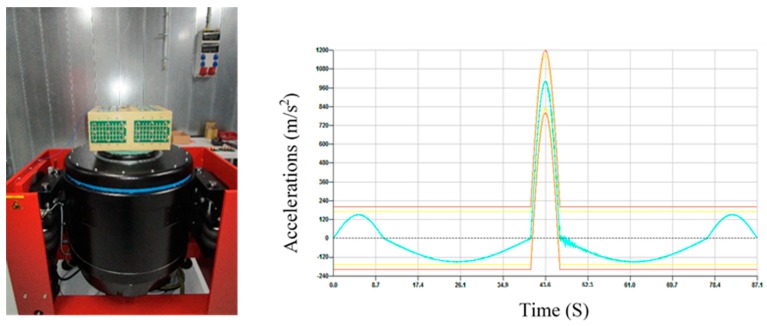
Mechanical Shock on PCB. (**left**) Testing equipment (**right**) Mechanical shock profile where the green line indicates the mechanical shock profile performed, and the other one shows the limits.

There were no falling components from the PCB’s, which shows strong durability of the six different solder pastes. Figure 14 shows the graph for the mechanical shock that the PCB’s went through.

### 3.5. Electrical Resistivity Measurement 

Since this research work was directed to an industrial application problem for electrical components, it is very necessary to evaluate the effect of the joints on the electrical resistivity. Therefore, in order to check how the soldering with and without Sn nanoparticle reinforcements may affect the current in the component after soldering, the RDC test was made on all components. A multimeter was used to see the difference between the solder pastes on the components or if there were any short circuits. It is known in the field that tin has better electrical conductivity than gold and it needs less time under the reflow oven to melt; hence, it has been widely used for its solderability in electrical components. For the RDC testing, 60 soldered components were tested for impedance from each solder paste type. 

**Figure 15:**
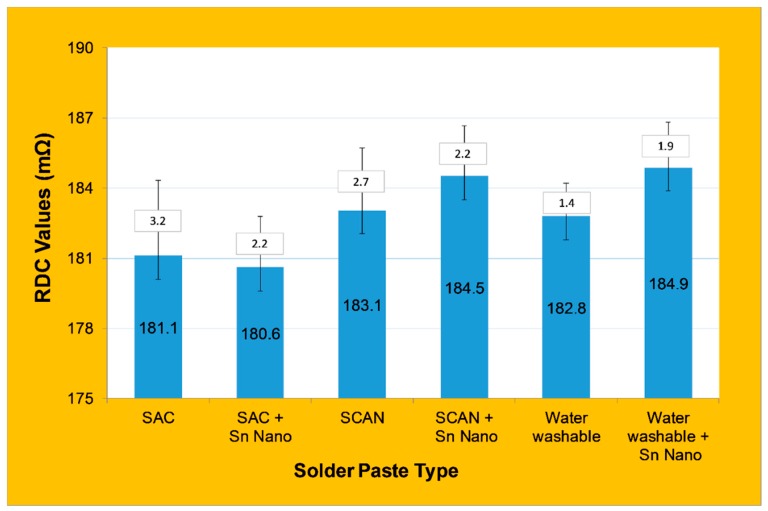
RDC values for components with different solder pastes.

The increase in the electrical component resistivity indicates a reduction in the conductivity; Figure 15 shows the RDC value for all solder pastes. The results show that the addition of Sn nanopowder resulted in different behavior of electrical resistivity, such that the RDC value was slightly reduced for the SAC solder paste but increased for SCAN and even more for the water washable solder pastes. Furthermore, it not only affected the average RDC value but also affected the deviation of the results, where it was reduced for SAC and SCAN and increased for water washable solder pastes. It is evident in Figure 15 that the SCAN with and without Sn nanopowder showed higher electrical resistivity (lower electrical conductivity), which is attributed to the Ni and Ge contents within the SCAN solder paste composition. The results show that the Sn nanopowder additions not only enhanced the strength as observed in the previous section but also resulted in better conductivity (lowest electrical resistivity) compared to all other types of solder pastes examined in this work. The measurement of electrical resistivity showed a unique behavior of SAC when the Sn nanopowder was added. The conductivity was increased, which indicates that the soldering was fully achieved and no voids were formed from this type of solder. Interestingly, from the microstructure analysis, it was noticed that homogenous joints were observed by SEM. Also, for the joint strength measurement, if we take the average of the highest strength between SAC and SAC with nanoparticles and compare the value to the other solders, we find that SAC has the highest maximum strength. This indicates that the soldering has the lowest amount of irregularities and voids, which allow electrical current to flow with higher electrical conductivity while maintaining the highest maximum strength. 

## 4. Conclusions

This study investigated the microstructure and joint strength of the WE-CNSW component on PCB using three different solder pastes with and without the addition of Sn nanopowder. The results showed that homogenous joint interfaces were observed for both SAC and SCAN solder pastes with and without nanopowder, where a less homogenized interface was observed in the water washable paste. On the other hand, the addition of Sn nanopowder affected the joint morphology, and it was speculated that joint formation kinetics occurred at different rates across the joint interface for SCAN and water washable solder pastes. CT scans revealed the existence of voids and bubbles within the joint interface, and it was higher when Sn nanopowder was used. All soldered samples showed high reliability when tested for mechanical shock and no falling components taken off from the PCB’s, which shows strong durability of the six different solder pastes. The addition of Sn nanopowder increased the variability in joint strength and resulted in the highest and lowest joint strength when Sn was added to SCAN solder pastes of 12.6 and 39.9 N. However, the addition of Sn nanopowder to SAC not only resulted in the highest average strength of 30 N but also reduced the resistance (increased electrical conductivity) as a result of forming a homogenous joint free of voids at the bond line. Based on this research work, we can identify a tendency, which will help us to define the next steps. Further tests with a higher sample size must be performed for a final validation for industrial application.

## Figures and Tables

**Table 1:** Chemical compositions for different solder pastes used.

Solder Paste-Type	Weight% (w.t.%)				
	Sn	Cu	Ag	Ni	Ge
SAC (SAC3-X(H)F3+-T3)	96.50	0.50	3.00	0.00	0.00
SCAN (SCAN-Ge071-XF3+-T3)	98.45	0.60	0.90	0.04	0.01
Water washable (P-AQ-SAC3-WW50-X121-T4)	96.50	0.50	3.00	0.00	0.00
